# PDE5 inhibitors enhance the lethality of pemetrexed through inhibition of multiple chaperone proteins and via the actions of cyclic GMP and nitric oxide

**DOI:** 10.18632/oncotarget.13640

**Published:** 2016-11-26

**Authors:** Laurence Booth, Jane L. Roberts, Andrew Poklepovic, Sarah Gordon, Paul Dent

**Affiliations:** ^1^ Department of Biochemistry and Molecular Biology, Virginia Commonwealth University, Richmond, VA 23298-0035, USA; ^2^ Department of Medicine, Virginia Commonwealth University, Richmond, VA 23298-0035, USA

**Keywords:** sildenafil, autophagy, pemetrexed, chaperone, lung cancer

## Abstract

Phosphodiesterase 5 (PDE5) inhibitors prevent the breakdown of cGMP that results in prolonged protein kinase G activation and the generation of nitric oxide. PDE5 inhibitors enhanced the anti-NSCLC cell effects of the NSCLC therapeutic pemetrexed. [Pemetrexed + sildenafil] activated an eIF2α – ATF4 – CHOP – Beclin1 pathway causing formation of toxic autophagosomes; activated a protective IRE1 – XBP-1 – chaperone induction pathway; and activated a toxic eIF2α – CHOP – DR4 / DR5 / CD95 induction pathway. [Pemetrexed + sildenafil] reduced the expression of c-FLIP-s, MCL-1 and BCL-XL that was blocked in a cell-type -dependent fashion by either over-expression of HSP90 / GRP78 / HSP70 / HSP27 or by blockade of eIF2α-CHOP signaling. Knock down of PKGI/II abolished the ability of sildenafil to enhance pemetrexed toxicity whereas pan-inhibition of NOS using L-NAME or knock down of [iNOS + eNOS] only partially reduced the lethal drug interaction. Pemetrexed reduced the ATPase activities of HSP90 and HSP70 in an ATM-AMPK-dependent fashion that was enhanced by sildenafil signaling via PKGI/II. The drug combination activated an ATM-AMPK-TSC2 pathway that was associated with reduced mTOR S2448 and ULK-1 S757 phosphorylation and increased ULK-1 S317 and ATG13 S318 phosphorylation. These effects were prevented by chaperone over-expression or by expression of an activated form of mTOR that prevented autophagosome formation and reduced cell killing. In two models of NSCLC, sildenafil enhanced the ability of pemetrexed to suppress tumor growth. Collectively we argue that the combination of [pemetrexed + PDE5 inhibitor] should be explored in a new NSCLC phase I trial.

## INTRODUCTION

Sorafenib, in addition to being an inhibitor of protein kinases was also recently discovered to be a potent inhibitor of chaperone ATPase activities that are associated with conformational changes in the ATP binding NH_2_-termini of the chaperone proteins and with the abilities of these chaperones to associate and co-localize with other chaperones as well as client proteins [1, 2]. It was reported that cGMP/PKG phosphorylation of chaperones inactivates their ATPase activities [3, 4]. More recently, it was shown that that PDE5 inhibitors such as sildenafil (Viagra) did not significantly alter basal chaperone ATPase activities but instead facilitated the multi-kinase inhibitor drugs sorafenib, regorafenib and pazopanib to cause further inhibition of chaperone ATPase activities and a more rapid NH_2_-terminus conformational change [1, 2, 5–9].

The changes in chaperone ATPase activity and conformation after [regorafenib + sildenafil], [sorafenib + sildenafil] and [pazopanib + sildenafil] exposure in prior publications were also reflected in the phosphorylation / activity of key chaperoned proteins [1, 2]. The multi-kinase inhibitor drugs interacted with sildenafil to inactivate the chaperone GRP78 that was associated with a large increase in PERK auto-phosphorylation and with subsequent eIF2α phosphorylation. Elevated eIF2α signaling increased the expression of the autophagy regulatory protein Beclin1. The drugs also interacted with sildenafil to inactivate HSP90 and HSP70 which in turn lost their ability to associate with HSP27. Combined loss of GRP78 and HSP27 function reduced signaling through the PI3 kinase pathway, causing mTOR inactivation. Reduced mTOR activity resulted in ULK-1 S757 phosphorylation declining and the phosphorylation of the ULK-1 substrate, ATG13 S318, becoming elevated. Phospho-ATG13 S318 was localized in autophagosomes with Beclin1. Knock down of ULK-1, ATG13 or Beclin1 prevented autophagosome formation and killing by these drug combinations. Over-expression of the chaperones GRP78 and HSP27 both prevented PERK activation and mTOR inactivation resulting in less autophagosome formation and less cell killing [1, 2].

Phosphodiesterase 5 inhibitors (PDE5 inhibitors) are used to treat erectile dysfunction [10]. PDE5 is also expressed in the wider vasculature and myocardium [11]. Tumor cells can over-express PDE5, as has been demonstrated in hepatoma, breast and NSCLC [7, 9]. PDE5 inhibitors have a well-established safety record and have been shown to be safe in combination with most other medications [12, 13]. The vast majority of studies examining the molecular biology of PDE5 inhibitors have been performed in vascular smooth muscle cells, monocytes and cardiac tissue; not in tumor cells. PDE5 catalyzes the degradation of cyclic GMP (cGMP); i.e. thus PDE5 inhibitors increase cGMP levels [reviewed in 14]. The second messenger nitric oxide (NO) induces smooth muscle relaxation via the actions of cGMP [15–18]. NO at nanomolar levels binds tightly to a heme group in NO-guanylyl cyclase (GC), also known as soluble guanylyl cyclase, and causes a ~150-fold activation of the enzyme. Activation of NO-GC elevates cGMP levels, which initiates the cGMP signaling pathway, in part through activation of cGMP dependent protein kinase (PKG) [19, 20].

It is known in non-tumor cells that cGMP/PKG, through its stimulatory actions upon the ERK1/2, p38 MAPK, JNK1/2 and NFκB pathways can increase the expression of inducible nitric oxide synthase (iNOS) [21–23]. Thus increased levels of NO activate GC and increase cGMP levels, that activates signaling pathways which increase iNOS levels; and, increased iNOS levels lead to further increases in cellular NO. i.e. potentially a self-stimulatory pathway. One mechanism by which NO is inactivated is by its reaction with the superoxide anion (O_2_^−^) [24, 25]. Compared to non-transformed cells, tumor cells generate greater amounts of O_2_^−^. The reaction of NO with O_2_^−^ forms the more potent oxidant peroxynitrite (ONOO^−^) that causes damaging S-nitrosylation of proteins and lipids [26–30].

The present studies were designed to determine the molecular mechanisms by which PDE5 inhibitors such as sildenafil (Viagra) enhance the anti-tumor effects of the standard of care drug pemetrexed in non-small cell lung cancer (NSCLC).

## RESULTS

**Figure 1:**
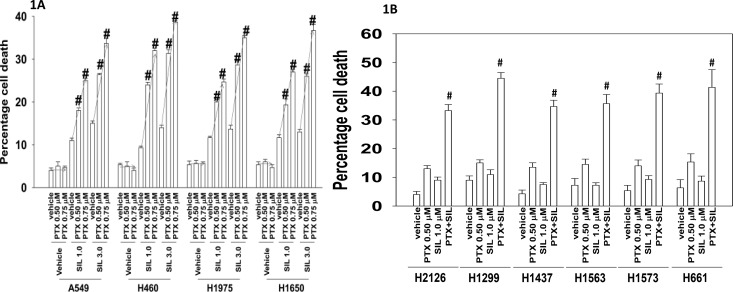
[Pemetrexed and Sildenafil] interact to kill tumor cells **A.** NSCLC cells were treated with the indicated concentrations of pemetrexed and sildenafil for 24h. Cells were isolated and viability determined by trypan blue exclusion assay (n = 3 +/− SEM) # p < 0.05 greater than corresponding value in vehicle treated cells. **B.** NSCLC cells were treated with the indicated concentrations of pemetrexed and sildenafil for 24h. Cells were isolated and viability determined by trypan blue exclusion assay (n = 3 +/− SEM) # p < 0.05 greater than corresponding value in pemetrexed treated cells.

**Figure 2:**
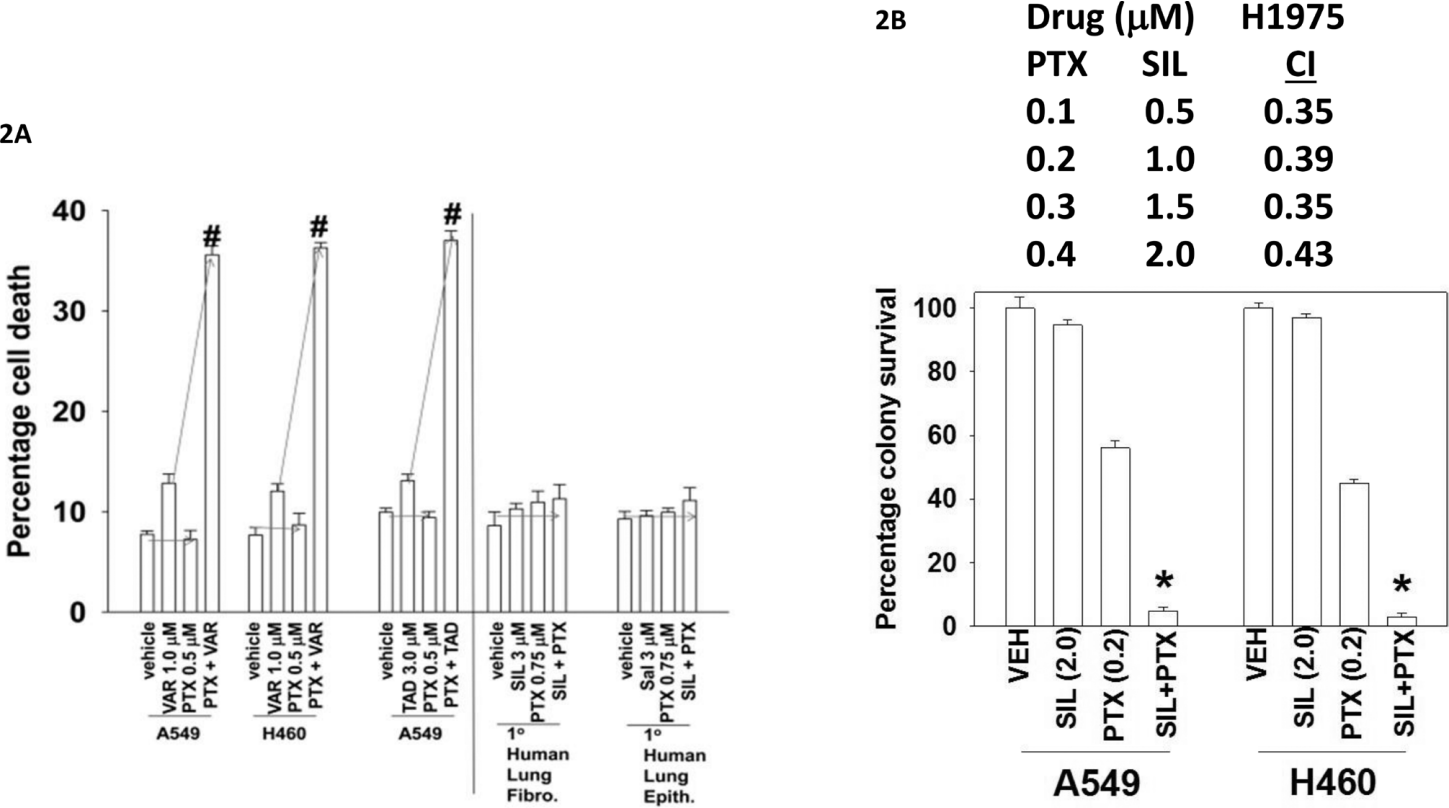
Tadalafil and vardenafil also selectively enhance pemetrexed lethality in tumor cells **A.** Lung cancer cells as well as primary lung fibroblasts and epithelial cells were treated with the indicated concentrations of pemetrexed, vardenafil, tadalafil and sildenafil, as indicated in the panel. Twenty-four h after treatment cells were isolated and viability determined by trypan blue exclusion assay (n = 3 +/− SEM) # p < 0.05 greater than the value in pemetrexed treated cells. **B.** NSCLC cells were plated in sextuplicate as single cells in 100 mm dishes (250, 500, 1,000 cells per dish). Twenty-four h after plating cells were treated for 12h with vehicle control, sildenafil, pemetrexed or the drugs in combination as indicated, at the indicated concentrations. After 12h the media was removed, the cells washed and fresh media added that did not contain drugs. Upper: Ten days after treatment cells were fixed in place, stained with crystal violet, and the number of colonies counted, with determination of changes in plating efficiency. The combination index (CI) for synergy was determined using the Calcusyn for Windows program. (n = 2 in sextuplicate +/− SEM). Lower: Ten days after treatment cells were fixed in place, stained with crystal violet, and the number of colonies counted, with determination of changes in plating efficiency (n = 2 in sextuplicate +/− SEM). * p < 0.05 lower than the value in pemetrexed treated cells.

Initial studies determined whether pemetrexed and sildenafil interacted to cause enhanced cell killing. To varying degrees, sildenafil enhanced the killing potential of pemetrexed in a genetically diverse range of lung cancer cells (Figure 1A and 1B). Similar data were obtained using other clinically relevant PDE5 inhibitors, tadalafil and vardenafil; the combination did not kill non-transformed cells in the same time-frame (Figure 2A). After a transient exposure, pemetrexed and sildenafil interacted in a synergistic fashion to kill lung cancer cells in colony formation assays (Figure 2B).

**Figure 3:**
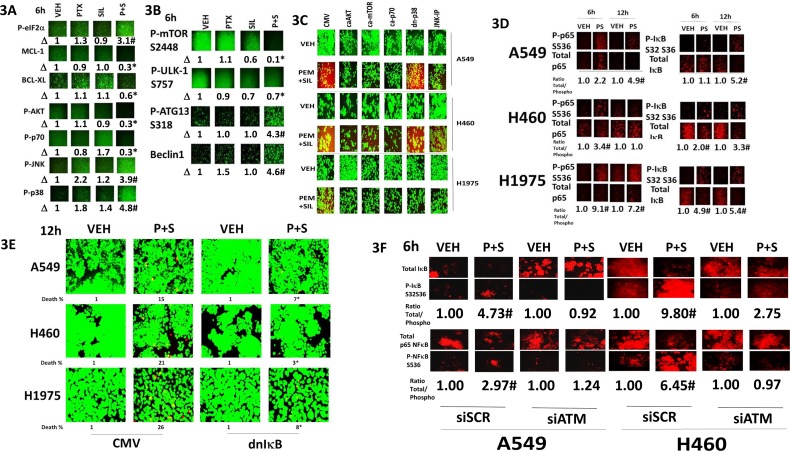
[Pemetrexed + sildenafil] inactivates the PI3K pathway and activates the JNK pathway that regulates tumor cell survival **A** and **B.** H460 cells were treated with vehicle control, pemetrexed (1.0 μM), sildenafil (2 μM) or the drugs in combination for 6h. Cells were fixed in place and immuno-fluorescence staining performed to determine the phosphorylation and expression of the indicated proteins (n = 3 +/− SEM) # p < 0.05 greater than pemetrexed alone value; * p < 0.05 less than vehicle control value. **C.** NSCLC cells were transfected with an empty vector plasmid (CMV) or with plasmids to express activated forms of AKT, mTOR or p70 S6K, or express dominant negative p38 MAPK. A portion of cells were transfected with empty vector plasmid and 30 min before drug exposure treated with the JNK inhibitory peptide (10 μM). Twenty-four h after transfection cells were treated with vehicle control, pemetrexed (1.0 μM), sildenafil (2 μM) or the drugs in combination for 24h. Floating cells were cytospun onto the 96 well plate and cell viability determined using a live / dead viability stain. **D.** NSCLC cells were treated with vehicle control or with [pemetrexed (1.0 μM), sildenafil (2 μM)] in combination for 6h. Cells were fixed in place and immuno-fluorescence staining performed to determine the phosphorylation and expression of the indicated proteins. (n = 3 +/− SEM) # p < 0.05 greater than vehicle control value. **E.** NSCLC cells were transfected with an empty vector plasmid (CMV) or with a plasmid to express the super-repressor IκB S32A S36A. Twenty-four h after transfection cells were treated with vehicle control, pemetrexed (1.0 μM), sildenafil (2 μM) or the drugs in combination for 24h. Floating cells were cytospun onto the 96 well plate and cell viability determined using a live / dead viability stain. (n = 3 +/− SEM) # p < 0.05 less than value in CMV transfected cells. **F.** NSCLC cells were transfected with a scrambled siRNA or with an siRNA to knock down ATM. Twenty-four h after transfection cells were treated with vehicle control or [pemetrexed (1.0 μM) + sildenafil (2 μM)] in combination for 6h. Cells were fixed in place and immuno-fluorescence staining performed to determine the phosphorylation and expression of the indicated proteins. (n = 3 +/− SEM) # p < 0.05 greater than vehicle control value.

Combined, but not individual, treatment of lung cancer cells strongly increased the phosphorylation of eIF2α S51, ATG13 S318, JNK and p38 whereas it decreased the phosphorylation of AKT T308, p70 S6K T389, mTOR S2448 and ULK-1 S757 (Figure 3A and 3B). These changes in phosphorylation were associated with reduced expression of BCL-XL and MCL-1, and increased expression of Beclin1. Expression of activated forms of AKT, mTOR or p70 S6K significantly reduced cell killing by [pemetrexed + sildenafil], as did inhibition of JNK pathway signaling (Figure 3C). Inhibition of p38 MAPK signaling did not alter the lethality of the drug combination. [Pemetrexed + sildenafil] treatment increased the phosphorylation of IκB and NFκB and reduced total IκB expression (Figure 3D). Blockade of NFκB signaling by expressing the super-repressor IκB S32A S36A *suppressed* [pemetrexed + sildenafil] lethality (Figure 3E). Pemetrexed, as a thymidylate synthase inhibitor, causes DNA damage which will activate the ataxia telangiectasia (ATM) protein [2]. The kinase ATM that can signal through IKKγ (NEMO) to activate NFκB; the drug-induced changes in NFκB and IκB phosphorylation as well as expression were dependent on ATM signaling (Figure 3F).

**Figure 4:**
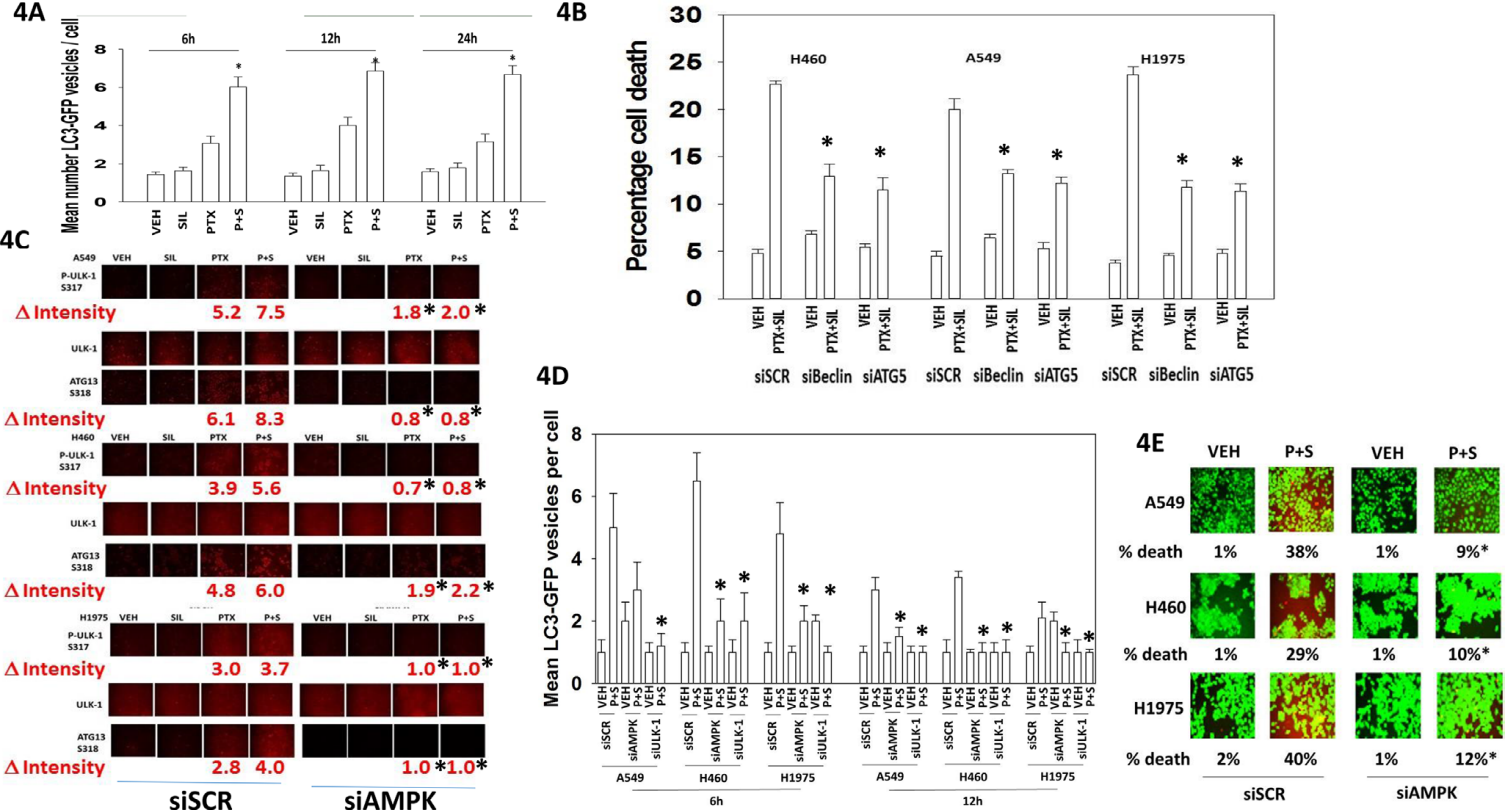
Pemetrexed-AMPK-ULK1 signaling is essential for the induction of toxic autophagy **A.** H460 cells were transfected with a plasmid to express LC3-GFP. Twenty-four h after transfection cells were treated with vehicle control, pemetrexed (1.0 μM), sildenafil (2 μM) or the drugs in combination for 6h, 12h or 24h. At each time point the cells were visualized and the number of intense punctate LC3-GFP bodies in each cell counted, with data acquired from at least 40 cells per condition, and the mean number of punctae per cell determined (n = 3 +/− SEM) * p < 0.05 greater than corresponding PTX alone value. **B.** NSCLC cells were transfected with a scrambled siRNA control (siSCR) or transfected to knock down the expression of ATG5 or Beclin1. Twenty-four h after transfection cells were treated with vehicle control or [pemetrexed (1.0 μM) + sildenafil (2 μM)] for 24h. Twenty-four h after treatment cells were isolated and tumor cell viability determined by trypan blue exclusion assay (n = 3 +/− SEM) * p < 0.05 less than the corresponding value in siSCR cells. **C.** NSCLC cells were transfected with a scrambled siRNA control (siSCR) or transfected to knock down the expression of AMPKα. After 24h, cells were treated with vehicle control, pemetrexed (1.0 μM), sildenafil (2 μM) or the drugs in combination for 6h. Cells were fixed in place and immuno-fluorescence staining performed to determine the phosphorylation and expression of the indicated proteins. (n = 3 +/− SEM) * p < 0.05 less than the corresponding value in siSCR cells. **D.** NSCLC cells were transfected with a scrambled siRNA control (siSCR) or transfected to knock down the expression of AMPKα; or ULK-1. After 24h, cells were treated with vehicle control or [pemetrexed (1.0 μM) + sildenafil (2 μM)] in combination for 6h or 12h. At each time point the cells were visualized and the number of intense punctate LC3-GFP bodies in each cell counted, with data acquired from at least 50 cells per condition, and the mean number of punctae per cell determined (n = 3 +/− SEM) * p < 0.05 less than corresponding value in siSCR cells. **E.** NSCLC cells were transfected with a scrambled siRNA control (siSCR) or transfected to knock down the expression of AMPKα. After 24h, cells were treated with vehicle control or [pemetrexed (1.0 μM) + sildenafil (2 μM)] in combination for 24h. Floating cells were cytospun onto the 96 well plate and viability determined using a live / dead viability stain where green cells are viable and yellow / red cells are dead (n = 3 +/− SEM) * p < 0.05 less than the corresponding value in siSCR cells.

In agreement with the drug combination causing elevated levels of Beclin1 and increased phosphorylation of ATG13 S318; increased numbers of autophagosomes were also detected in cells treated with [pemetrexed + sildenafil] (Figure 4A). Knock down of Beclin1 or ATG5 reduced the lethality of [pemetrexed + sildenafil] treatment (Figure 4B). Pemetrexed, via elevating ZMP levels, promotes activation of the AMP-dependent kinase (AMPK) [31, 32]. The AMPK phosphorylates ULK-1 on S317 which causes ULK-1 activation [33]. Pemetrexed, and to a greater extent [pemetrexed + sildenafil], increased both ULK-1 S317 and ATG13 S318 phosphorylation in an AMPK-dependent manner (Figure 4C). Thus for ATG13 phosphorylation and hence autophagosome formation to occur requires ULK-1 S757 dephosphorylation *and* increased ULK-1 S317 phosphorylation. In agreement with our ULK-1 S317 data, knock down of AMPK also significantly reduced the ability of [pemetrexed + sildenafil] to increase autophagosome levels and to cause tumor cell death (Figure 4D and 4E).

**Figure 5:**
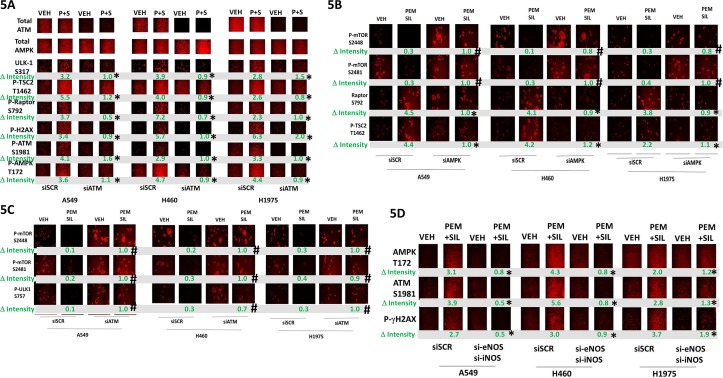
Pemetrexed-induced DNA damage / ATM signaling facilitates activation of the AMPK **A.** NSCLC cells were transfected with a scrambled siRNA control (siSCR) or transfected to knock down the expression of ATM. After 24h, cells were treated with vehicle control, pemetrexed (1.0 μM), sildenafil (2 μM) or the drugs in combination for 6h. Cells were fixed in place and immuno-fluorescence staining performed to determine the phosphorylation and expression of the indicated proteins. (n = 3 +/− SEM) * p < 0.05 less than the corresponding value in siSCR cells. **B.** NSCLC cells were transfected with a scrambled siRNA control (siSCR) or transfected to knock down the expression of AMPKα. After 24h, cells were treated with vehicle control, pemetrexed (1.0 μM), sildenafil (2 μM) or the drugs in combination for 6h. Cells were fixed in place and immuno-fluorescence staining performed to determine the phosphorylation and expression of the indicated proteins. (n = 3 +/− SEM) # p < 0.05 greater than the corresponding value in siSCR cells; * p < 0.05 less than the corresponding value in siSCR cells. **C.** NSCLC cells were transfected with a scrambled siRNA control (siSCR) or transfected to knock down the expression of ATM. After 24h, cells were treated with vehicle control, pemetrexed (1.0 μM), sildenafil (2 μM) or the drugs in combination for 6h. Cells were fixed in place and immuno-fluorescence staining performed to determine the phosphorylation and expression of the indicated proteins. (n = 3 +/− SEM) # p < 0.05 greater than the corresponding value in siSCR cells. **D.** NSCLC cells were transfected with a scrambled siRNA control (siSCR) or transfected to knock down the expression of iNOS and eNOS together. After 24h, cells were treated with vehicle control, pemetrexed (1.0 μM), sildenafil (2 μM) or the drugs in combination for 6h. Cells were fixed in place and immuno-fluorescence staining performed to determine the phosphorylation and expression of the indicated proteins. (n = 3 +/− SEM) * p < 0.05 less than the corresponding value in siSCR cells.

Pemetrexed causes DNA damage which will activate ATM [2]. Sildenafil, through generation of nitric oxide would also be predicted to activate ATM. Treatment of cells with [pemetrexed + sildenafil] activated ATM as judged by increased phosphorylation of ATM itself and histone γ2AX, and the lack of γH2AX phosphorylation when ATM expression was knocked down (Figure 5A). ATM has been proposed to phosphorylate the AMPK on T172 and TSC2 on T1462. In an ATM-dependent fashion, [pemetrexed + sildenafil] increased the phosphorylation of AMPK T172, Raptor S792 and TSC2 T1462. These findings correlated with increased ULK-1 S317 phosphorylation. In an AMPK-dependent fashion, [pemetrexed + sildenafil] increased phosphorylation of Raptor S792 and TSC2 T1462 and decreased the phosphorylation of mTOR S2448 and S2481 (Figure 5B). The drug-induced decreased phosphorylation of mTOR S2448, mTOR S2481 and ULK-1 S757 was prevented by knock down of ATM (Figure 5C). To our surprise, knock down of either ATM or of AMPK also increased the basal phosphorylation levels of mTOR S2448, mTOR S2481 as well as of their downstream target ULK-1 S757. Low levels of nitrosative stress have been shown to activate the ATM-AMPK pathway in an LKB-1 -dependent fashion, however as A549 and H460 cells do not express LKB-1 this argues in our drug combination system LKB-1 is dispensable for AMPK activation which may be due to pemetrexed increasing the levels of ZMP that will allosterically activate the enzyme. One other possible reason for this may be that in our system ATM activation is induced by both pemetrexed-induced DNA damage and by sildenafil-induced nitric oxide. To test whether nitric oxide signaling facilitated ATM and AMPK phosphorylation we knocked down the expression of [iNOS + eNOS]. Inhibition of nitric oxide generation reduced by ~50% to 100% the ability of [pemetrexed + sildenafil] to increase ATM and AMPK phosphorylation (Figure 5E). Collectively these findings imply that [pemetrexed + sildenafil] induces an ATM-AMPK signaling module which, through multiple overlapping mechanisms, reduces mTOR activity and increases ULK-1 activity, leading to toxic autophagosome formation.

**Figure 6:**
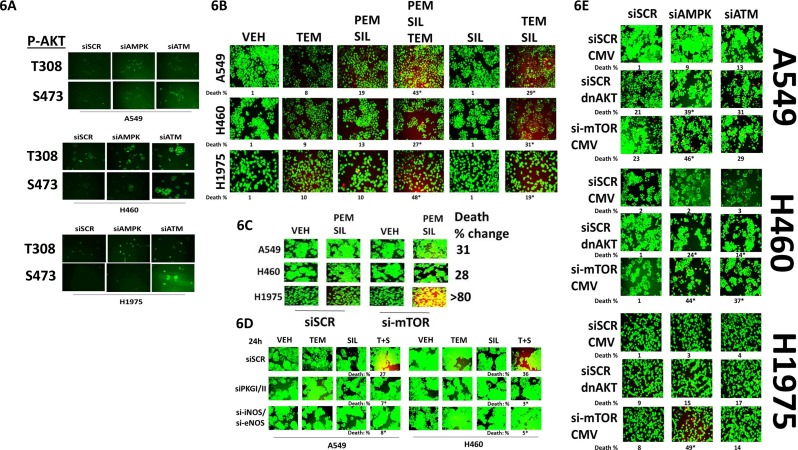
AKT / mTOR signaling is protective against [pemetrexed + sildenafil] **A.** NSCLC cells were transfected with a scrambled siRNA control (siSCR) or transfected to knock down the expression of ATM or of AMPKα. After 24h, cells were fixed in place and immuno-fluorescence staining performed to determine the phosphorylationof AKT T308 and AKT S473. **B.** NSCLC cells were treated with vehicle control or [pemetrexed (1.0 μM) + sildenafil (2 μM)] and/or Temsirolimus (0.5 μM) in combination as indicated for 24h. Floating cells were cytospun onto the 96 well plate and viability determined using a live / dead viability stain where green cells are viable and yellow / red cells are dead (n = 3 +/− SEM) * p < 0.05 less than the corresponding value in siSCR cells. **C.** NSCLC cells were transfected with a scrambled siRNA or were transfected to knock down the expression of mTOR. After 24h, cells were treated with vehicle control or with [pemetrexed 1.0 μM + sildenafil 2.0 μM]. After a further 24h floating cells were cytospun onto the 96 well plate and viability determined using a live / dead viability stain where green cells are viable and yellow / red cells are dead (n = 3 +/− SEM) * p < 0.05 less than the corresponding value in siSCR cells. **D.** NSCLC cells were transfected with a scrambled siRNA control or were transfected to knock down expression of ATM. After 24h, cells were treated with vehicle control or with [Temsirolimus 0.5 μM + sildenafil 2.0 μM]. After a further 24h floating cells were cytospun onto the 96 well plate and viability determined using a live / dead viability stain where green cells are viable and yellow / red cells are dead (n = 3 +/− SEM) * p < 0.05 less than the corresponding value in siSCR cells. **E.** NSCLC cells were transfected with an empty vector plasmid or a scrambled siRNA control or were transfected to express dominant negative AKT or knock down expression of mTOR and/or to knock down the expression of ATM or of AMPKα. After 24h, floating cells were cytospun onto the 96 well plate and viability determined using a live / dead viability stain where green cells are viable and yellow / red cells are dead (n = 3 +/− SEM) * p < 0.05 less than the corresponding value in siSCR cells.

In addition to our findings in Figure 5, the phosphorylation of AKT T308 and AKT T473 was also variably enhanced by knock down of ATM or of AMPK (Figure 6A). Treatment of NSCLC cells with a clinically relevant concentration of the mTOR inhibitor temsirolimus enhanced the lethality of [pemetrexed + sildenafil] (Figure 6B). Of note, sildenafil and temsirolimus also interacted in a greater than additive fashion to kill tumor cells. Knock down of mTOR also enhanced the lethality of [pemetrexed + sildenafil] (Figure 6C). Knock down of PKG or of iNOS/eNOS prevented sildenafil interacting with temsirolimus to kill tumor cells (Figure 6D). In Figure 5 we found that knock down of ATM or of AMPK modestly enhanced the activities of mTOR and AKT. Expression of dominant negative AKT weakly and variably enhanced cell death in tumor cells transfected to knock down the expression of ATM or AMPK (Figure 6E). In contrast, knock down of mTOR enhanced cell death in tumor cells transfected to knock down the expression of AMPK, and to a lesser extent than that caused by ATM knock down (Figure 6E).

**Figure 7:**
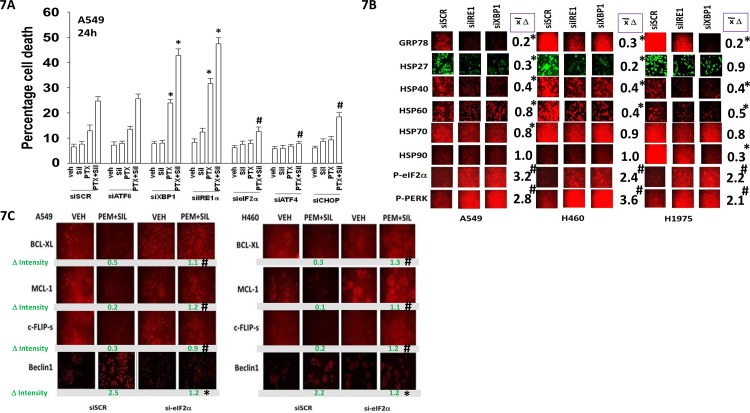
Endoplasmic reticulum stress signaling regulates the ability of [pemetrexed + sildenafil] to kill tumor cells by altering the expression of chaperone proteins and cyto-protective proteins **A.** A549 cells were transfected with a scrambled siRNA control (siSCR) or transfected to knock down the expression of: ATF6, XBP-1, IRE1, eIF2α, ATF4 or CHOP. Twenty-four h after transfection cells were treated with vehicle control, pemetrexed (1.0 μM), sildenafil (2 μM) or the drugs in combination for 24h. Cells were then isolated and viability determined by trypan blue exclusion assay (n = 3 +/− SEM) # p < 0.05 less than corresponding value in siSCR transfected cells; * p < 0.05 greater than corresponding value in siSCR transfected cells. **B.** NSCLC cells were transfected with a scrambled siRNA control (siSCR) or transfected to knock down the expression of XBP-1 or IRE1. Twenty-four h after transfection cells were fixed in place and immuno-fluorescence staining performed to determine the phosphorylation and expression of the indicated proteins. (n = 3 +/− SEM) # p < 0.05 greater than corresponding value in siSCR transfected cells; * p < 0.05 less than corresponding value in siSCR transfected cells. **C.** A549 and H460 cells were transfected with a scrambled siRNA control (siSCR) or transfected to knock down the expression of eIF2α. Twenty-four h after transfection cells were treated with vehicle control or [pemetrexed (1.0 μM) + sildenafil (2 μM)] in combination for 6h. Cells were then fixed in place and immuno-fluorescence staining performed to determine the phosphorylation and expression of the indicated proteins. (n = 3 +/− SEM) # p < 0.05 greater than corresponding value in siSCR transfected cells; * p < 0.05 less than corresponding value in siSCR transfected cells

As eIF2α was phosphorylated after drug combination exposure, and autophagosome formation enhanced, we next determined the relative importance of the known endoplasmic reticulum stress signaling pathways in the death and/or survival of tumor cells treated with [pemetrexed + sildenafil]. Knock down of eIF2α, ATF4 or CHOP reduced the lethality of [pemetrexed + sildenafil] treatment (Figure 7A). Knock down of ATF6 neither enhanced nor suppressed [pemetrexed + sildenafil] lethality. In contrast, knock down of IRE1 or XBP1 increased the lethality of pemetrexed and of [pemetrexed + sildenafil]. Knock down of IRE1 or XBP1 reduced expression of the cyto-protective chaperones GRP78, HSP27, HSP40, HSP60 and HSP70, with a cell-type dependent reduction in HSP90 levels (Figure 7B). The reduction in GRP78 levels was mirrored in the increased phosphorylation of PERK and eIF2α. Treatment of cells with [pemetrexed + sildenafil] reduced the expression of the cyto-protective proteins BCL-XL, MCL-1 and c-FLIP-s, an effect that was blocked by knock down of eIF2α (Figure 7C). In parallel the drug combination also increased expression of Beclin1 that was prevented by eIF2α knock down.

Over-expression of HSP90, HSP70, GRP78 and [HSP70 + GRP78], [HSP70 + HSP90] or [HSP70 + HSP27] significantly reduced the lethality of [pemetrexed + sildenafil] treatment (Supplementary Figure S1A). Notably the combinations of [HSP70 + GRP78] and [HSP70 + HSP27] in a cell-type dependent fashion were able to further reduce cell killing beyond that afforded by individual expression of the chaperones. Over-expression of chaperones increased the basal expression of Beclin1 and maintained Beclin1 expression after [pemetrexed + sildenafil] exposure (Supplementary Figure S1B). [Pemetrexed + sildenafil] treatment reduced the expression of BCL-XL, MCL-1 and c-FLIP-s that was preserved in a cell-type dependent fashion by over-expression of chaperones (Supplementary Figures S1C, S1D and S1E). As shown in Figure 7C, knock down of eIF2α prevented [pemetrexed + sildenafil] treatment reducing BCL-XL, MCL-1 and c-FLIP-s levels; over-expression of chaperones, alone or in combination, reduced the drug combination-induced phosphorylation of eIF2α (Supplementary Figure S1F). More noticeably were the differential effects on autophagy gate-keeper kinase mTOR phosphorylation, caused by chaperone over-expression; mTOR occurs in two protein complexes termed mTORC1 and mTORC2. Phosphorylation of mTOR at Serine 2448 is a biomarker for mTORC1 activity whereas phosphorylation of mTOR Serine 2481 is a biomarker for mTORC2 activity. Chaperone over-expression consistently rescued mTOR S2448 phosphorylation (mTORC1) from the inhibitory effects of [pemetrexed + sildenafil] exposure (Supplementary Figure S1F). However, chaperone over-expression in a cell type dependent manner only partially protected mTOR S2481 (mTORC2) phosphorylation.

**Figure 8:**
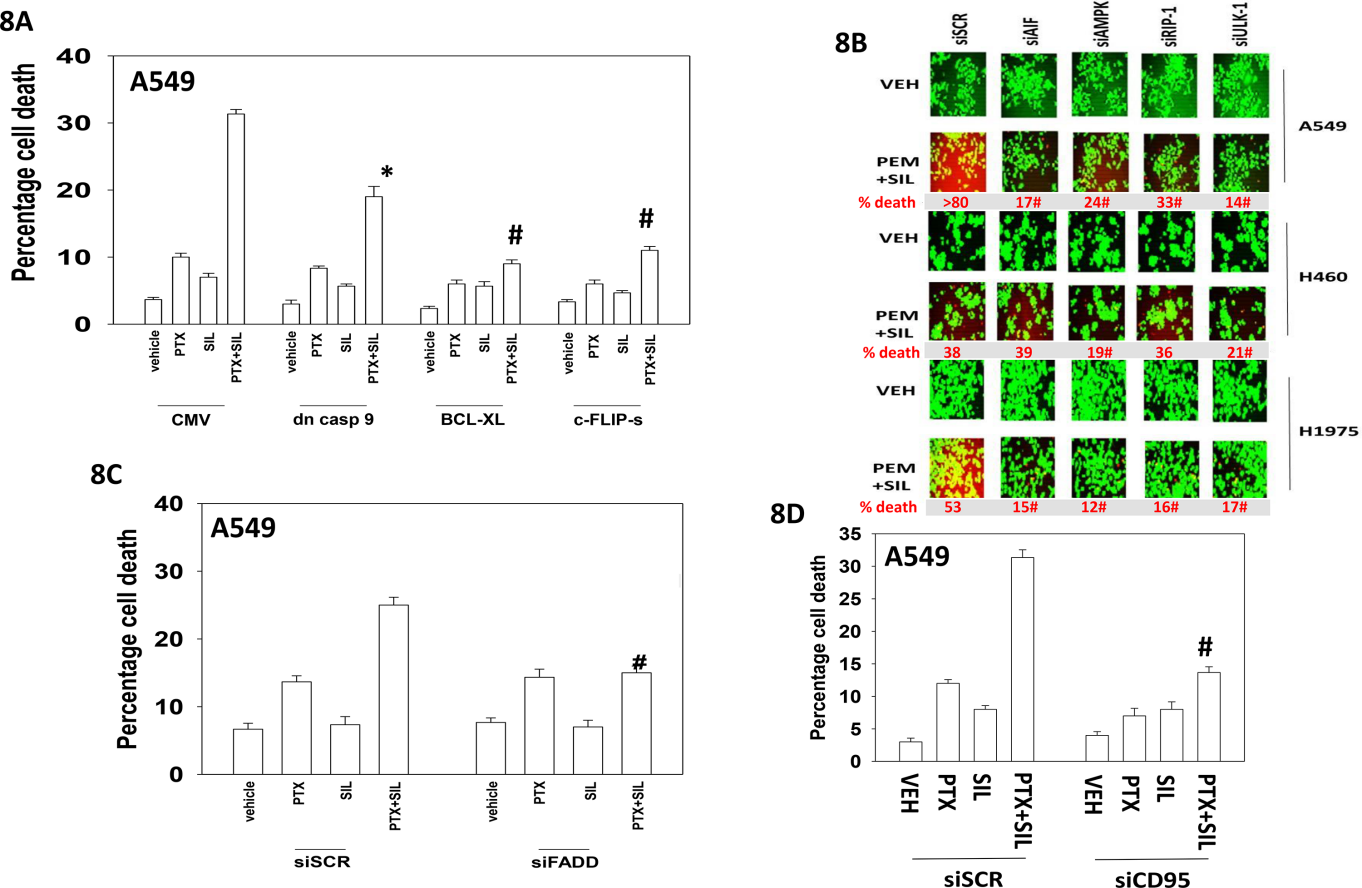
Necroptotic signaling downstream of CD95 plays a key role in [pemetrexed + sildenafil] toxicity **A.** A549 cells were transfected with an empty vector plasmid (CMV) or plasmids to express: dominant negative caspase 9; GRP78; BCL-XL; c-FLIP-s, as indicated. Twenty-four h after transfection cells were treated with vehicle control, pemetrexed (1.0 μM), sildenafil (2 μM)] or the drugs in combination for 24h. Cells were isolated and viability determined by trypan blue exclusion assay (n = 3 +/− SEM) * p < 0.05 less than corresponding value in CMV transfected cells; # p < 0.05 less than corresponding value in cells expressing dominant negative caspase 9. **B.** NSCLC cells were transfected with a scrambled siRNA control (siSCR) or transfected to knock down the expression of: AIF, AMPKα, RIP-1 or ULK-1. Twenty-four h after transfection cells were treated with vehicle control or [pemetrexed (1.0 μM) + sildenafil (2 μM)] in combination for 24h. Floating cells were cytospun onto the 96 well plate and viability determined using a live / dead viability stain where green cells are viable and yellow / red cells are dead. **C.** and **D.** A549 cells were transfected with a scrambled siRNA control (siSCR) or transfected to knock down the expression of FADD or of CD95. Twenty-four h after transfection cells were treated with vehicle control or [pemetrexed (1.0 μM) + sildenafil (2 μM)] in combination for 24h. Cells were isolated and viability determined by trypan blue exclusion assay (n = 3 +/− SEM) # p < 0.05 less than corresponding value in siSCR transfected cells.

**Figure 9:**
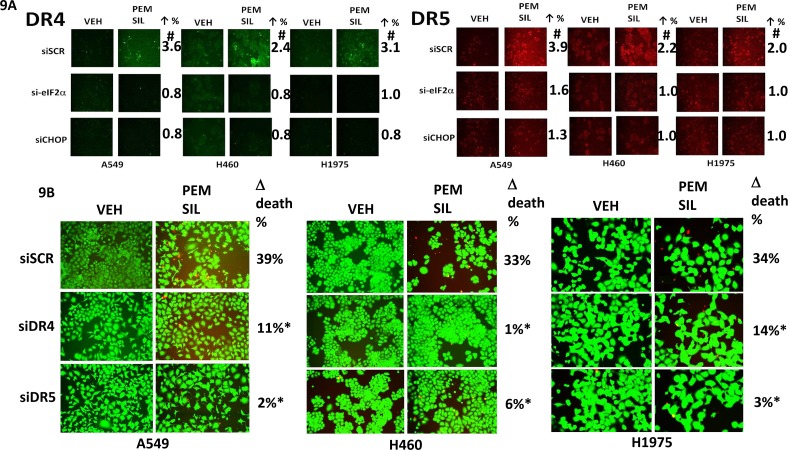
[Pemetrexed + sildenafil] treatment increases the expression of DR4 and DR5 via eIF2α-CHOP signaling and knock down of DR4/DR5 protects against drug combination toxicity **A.** NSCLC cells were transfected with a scrambled siRNA control (siSCR) or transfected to knock down the expression of eIF2α or of CHOP. Twenty-four h after transfection cells were treated with vehicle control or [pemetrexed (1.0 μM) + sildenafil (2 μM)] in combination for 6h. Cells were then fixed in place and immuno-fluorescence staining performed to determine the expression of DR4 and DR5. (n = 3 +/− SEM) # p < 0.05 greater than corresponding value in siSCR transfected cells. **B.** NSCLC cells were transfected with a scrambled siRNA control (siSCR) or transfected to knock down the expression of DR4 or of DR5. Twenty-four h after transfection cells were treated with vehicle control or [pemetrexed (1.0 μM) + sildenafil (2 μM)] in combination for 24h. Floating cells were cytospun onto the 96 well plate and viability determined using a live / dead viability stain where green cells are viable and yellow / red cells are dead (n = 3 +/−SEM) * p < 0.05 less than corresponding value in siSCR cells.

Additional modes of tumor cell killing were also interrogated for any possible role in drug combination lethality. Over-expression of a dominant negative caspase 9 protein significantly reduced [pemetrexed + sildenafil] lethality by ~50% whereas over-expression of BCL-XL or c-FLIP-s reduced killing by > 75% (Figure 8A). Knock down of apoptosis inducing factor (AIF) or of RIP-1 also significantly reduced killing by the drug combination in A549 and H1975 cells though did not alter killing in H460 cells (Figure 8B). Knock down of ULK-1 was uniformly protective. Knock down of the death receptor CD95 or the death receptor docking effector protein FADD significantly reduced the ability of sildenafil to enhance pemetrexed lethality (Figure 8C and 8D). Activation of eIF2α-CHOP signaling has also been linked to increased expression of the death receptors DR4 and DR5. Treatment of cells with [pemetrexed + sildenafil] increased DR4 and DR5 expression in an eIF2α-CHOP -dependent manner (Figure 9A). Knock down of DR4 or DR5 in a cell type dependent fashion suppressed the lethality of [pemetrexed + sildenafil] (Figure 9B).

**Figure 10:**
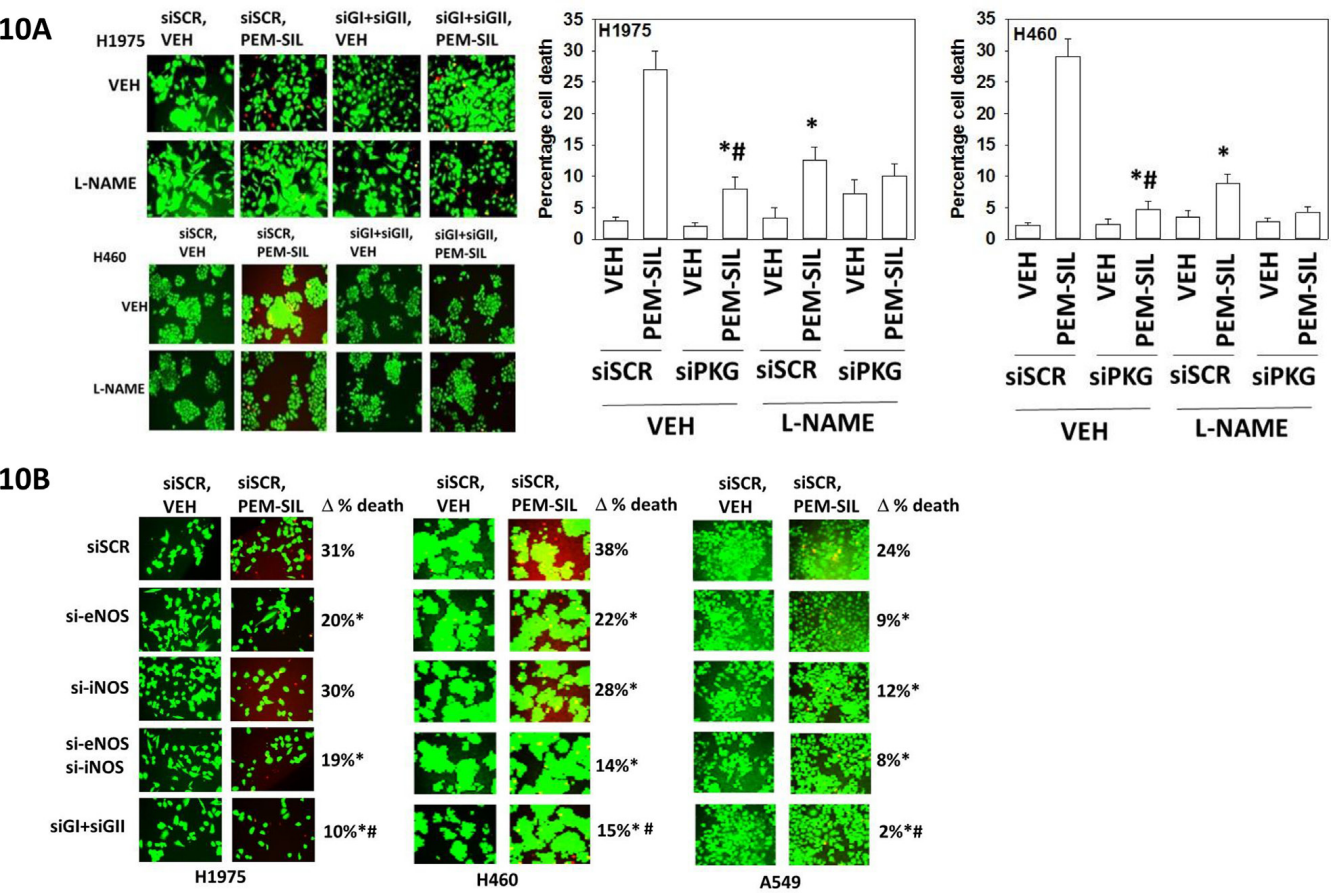
Sildenafil-induced PKG signaling plays a greater role in enhancing pemetrexed toxicity than does elevated nitric oxide synthase signaling **A.** H1975 and H460 cells were transfected with a scrambled siRNA control (siSCR) or transfected to knock down the expression of PKGI and PKGII. Twenty-four h after transfection cells were treated with vehicle control or [pemetrexed (1.0 μM) + sildenafil (2 μM)] in combination for 24h. Thirty minutes prior to drug treatment, cells were treated with vehicle control or with the nitric oxide synthase inhibitor L-NAME (1 μM). Floating cells were cytospun onto the 96 well plate and viability determined using a live / dead viability stain where green cells are viable and yellow / red cells are dead (n = 3 +/−SEM) * p < 0.05 less than corresponding value in siSCR cells; # p < 0.05 less than corresponding value in siSCR + L-NAME cells. **B.** NSCLC cells were transfected with a scrambled siRNA control (siSCR) or transfected to knock down the expression of: iNOS; eNOS; or PKGI and PKGII, as indicated. Twenty-four h after transfection cells were treated with vehicle control or [pemetrexed (1.0 μM) + sildenafil (2 μM)] in combination for 24h. Floating cells were cytospun onto the 96 well plate and viability determined using a live / dead viability stain where green cells are viable and yellow / red cells are dead (n = 3 +/−SEM) * p < 0.05 less than corresponding value in siSCR cells; # p < 0.05 less than values in individual NOS knock down cells.

The roles of cGMP and nitric oxide in the regulation of pemetrexed toxicity were next investigated. Combined knock down of PKGI and PKGII expression significantly reduced the ability of sildenafil to enhance pemetrexed toxicity (Figure 10A). Pan-inhibition of nitric oxide synthase (NOS) enzymes using L-NAME also significantly reduced the ability of sildenafil to enhance pemetrexed lethality although this effect was significantly less than that afforded by knock down of PKGI/II. We then determine which NOS enzymes were responsible for facilitating the sildenafil effect. We did not observe nNOS expression in our lung cancer cells. In H1975 cells knock down of eNOS, but not iNOS, reduced [pemetrexed + sildenafil] lethality (Figure 10B). In H460 and A549 cells, however, knock down of either iNOS or eNOS reduced drug combination lethality. Prior studies with the drugs AR12, sorafenib and pazopanib, alone or in combination with sildenafil, demonstrated that they reduce the ATPase activities of the HSP90 and HSP70 chaperone proteins which correlates with altered tertiary conformation and altered chaperone-client interactions [1, 2].

**Figure 11:**
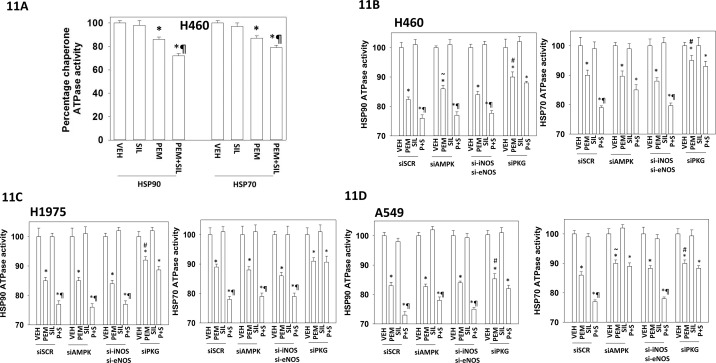
PKG-dependent basal and stimulated chaperone phosphorylation is required for the inhibition of HSP90 and HSP70 by [pemetrexed + sildenafil] **A.** H460 cells were transfected with a plasmid to express FLAG-HSP90 or with a plasmid to express HA-HSP70. Twenty-four h after transfection the cells were treated with vehicle control, pemetrexed (1.0 μM), sildenafil (2 μM)] or the drugs in combination for one hour. Cells were then lysed and HSP90 and HSP70 immuno-precipitated using their FLAG and HA tags. The ATPase activity of each chaperone was determined as described in the Methods (n = 3 +/− SEM) * p < 0.05 less than vehicle control; ¶p < 0.05 less than pemetrexed single agent value. **B-D.** NSCLC cells were transfected with a scrambled control siRNA (siSCR) or with siRNA molecules to knock down the expression of: AMPKα; iNOS and eNOS; PKGI and PKGII, as indicated. In parallel, cells were transfected with a plasmid to express FLAG-HSP90 or with a plasmid to express HA-HSP70. Twenty-four h after transfection the cells were treated with vehicle control or [pemetrexed (1.0 μM) + sildenafil (2 μM)] in combination for one hour. Cells were then lysed and HSP90 and HSP70 immuno-precipitated using their FLAG and HA tags. The ATPase activity of each chaperone was determined as described in the Methods (n = 3 +/− SEM) * p < 0.05 less than vehicle control; ¶p < 0.05 less than pemetrexed single agent value; # p < 0.05 greater than corresponding value in siSCR transfected cells.

**Figure 12:**
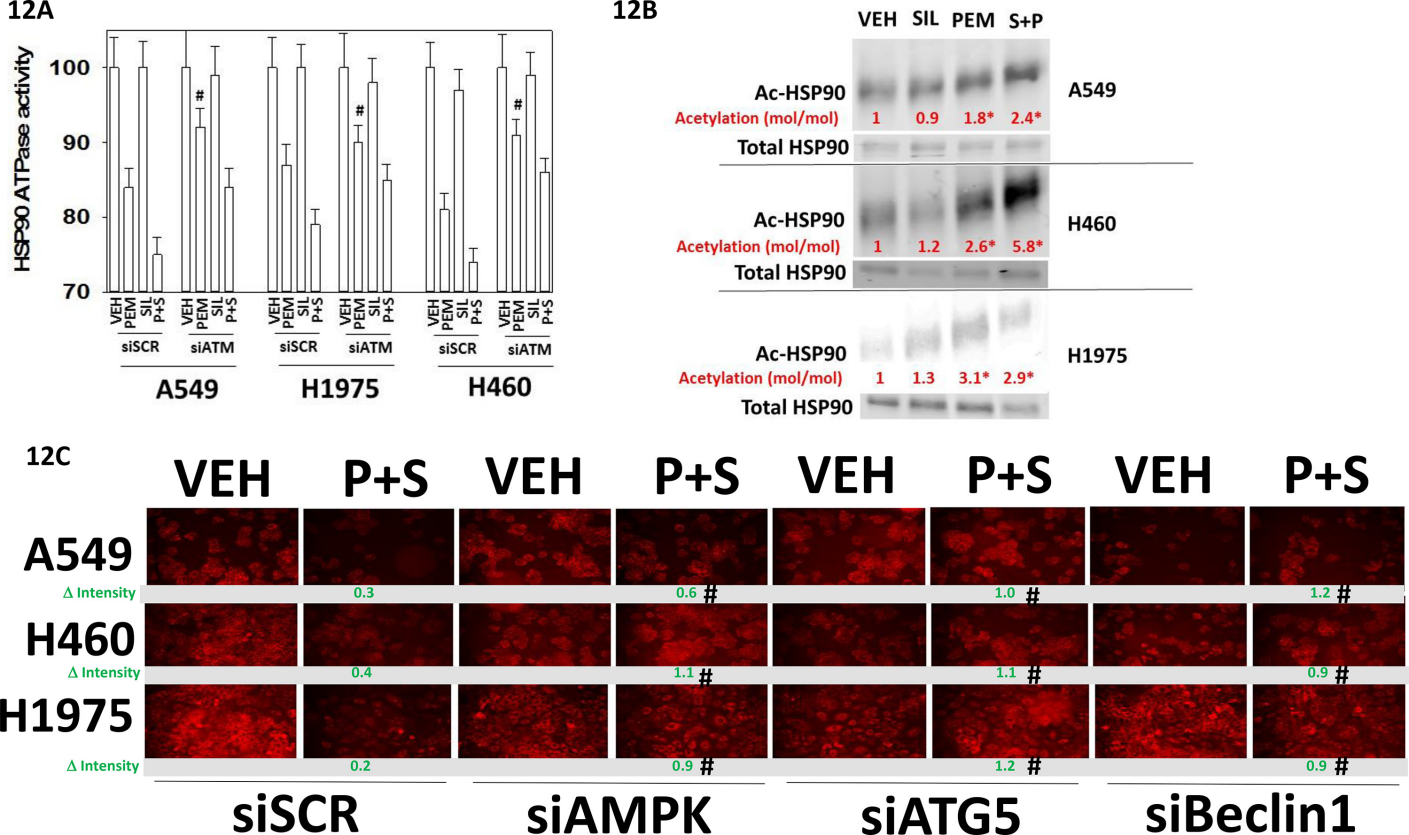
ATM signaling mediates the pemetrexed -induced inactivation of HSP90 **A.** NSCLC cells were transfected with a scrambled control siRNA (siSCR) or with an siRNA molecule to knock down the expression of ATM. In parallel, cells were transfected with a plasmid to express FLAG-HSP90. Twenty-four h after transfection the cells were treated with vehicle control, pemetrexed (1.0 μM), sildenafil (2 μM)] or the drugs in combination for one hour. Cells were then lysed and HSP90 immuno-precipitated using its FLAG tag. The ATPase activity of HSP90 was determined as described in the Methods (n = 3 +/− SEM) * p < 0.05 less than vehicle control; ¶p < 0.05 less than pemetrexed single agent value; # p < 0.05 greater than corresponding value in siSCR transfected cells. **B.** NSCLC cells were transfected with a plasmid to express FLAG-HSP90. Twenty-four h after transfection cells were treated with vehicle control, pemetrexed (1.0 μM), sildenafil (2 μM) or the drugs in combination for one hour. Cells were then lysed and HSP90 immuno-precipitated using its FLAG tag. Immuno-precipitates were subjected to SDS PAGE and immuno-blotting to determine total HSP90 expression; and HSP90 acetylation. The level of acetylation (moles of acetylated HSP90 / moles of total HSP90) was determined for each condition with the value in vehicle treated cells set/defined as 1.00 (n = 3 +/− SEM). * p < 0.05 greater acetylation level than in vehicle control cells. **C.** NSCLC cells were transfected with a scrambled control siRNA (siSCR) or with siRNA molecules to knock down the expression of AMPKα or Beclin1 or ATG5. Twenty-four h after transfection cells were treated with vehicle control, pemetrexed (1.0 μM), sildenafil (2 μM) or the drugs in combination for six hours. Cells were then fixed in place and immuno-fluorescence staining performed to determine the expression of HDAC6. (n = 3 +/− SEM) # p < 0.05 greater than corresponding value in siSCR transfected cells.

Treatment of cells with pemetrexed as a single agent followed by chaperone isolation reduced the *in vitro* ATPase activities of HSP90 and HSP70; an effect that was enhanced by sildenafil (Figure 11A). For HSP90 and HSP70 isolated from [pemetrexed + sorafenib] treated cells knock down of PKGI/II prevented sildenafil enhancing the chaperone inhibitory activity of pemetrexed whereas knock down of iNOS and eNOS had no effect (Figures 11B, 11C and 11D). Knock down of PKGI/II also significantly reduced the ability of pemetrexed to suppress chaperone activities suggesting that basal levels of PKG-mediated phosphorylation are required to prime the chaperones for the pemetrexed inhibitory effect. In H460 cells with HSP90 and in A549 cells for HSP70, knock down of ATM reduced the chaperone inhibitory effect of pemetrexed arguing that ATM-mediated inhibitory phosphorylation of the chaperones was in part a mechanism by which pemetrexed was reducing chaperone activity (Figure 12A). The activity of HSP90 can also be regulated by acetylation. Treatment of NSCLC cells with pemetrexed increased the acetylation of HSP90 (Figure 12B). In A549 and H460 cells the combination of [pemetrexed + sildenafil] caused more acetylation than pemetrexed alone (p < 0.05). HSP90 acetylation is regulated in part by HDAC6. Treatment of NSCLC cells for 6h with [pemetrexed + sildenafil] reduced the protein levels of HDAC6, which correlated with the increased levels of HSP90 acetylation we had observed (Figure 12B and 12C). Knock down of AMPKα or Beclin1 prevented [pemetrexed + sildenafil] from reducing HDAC6 expression, arguing that HDAC6 was being eliminated through a process involving autophagic digestion.

**Figure 13:**
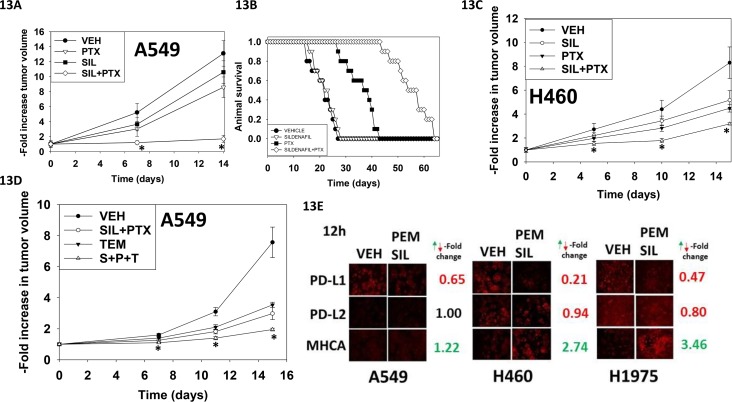
Sildenafil enhances the anti-tumor effects of pemetrexed *in vivo* **A.** A549 cells were implanted into the rear flank of male athymic mice (the line was isolated from a male patient). Tumors were permitted to form and the animals treated as described in the Methods section. * p < 0.05 less than pemetrexed single agent treatment. **B.** A Kaplan Meier survival curve was plotted using the data from Panel A; animals were humanely sacrificed based on approved IACUC protocols when the tumor volume reached 1,500 mm^3^. **C.** H460 cells into the rear flank of male athymic mice (the line was isolated from a male patient). Tumors were permitted to form and the animals treated as described in the Methods section. * p < 0.05 less than pemetrexed single agent treatment. **D.** A549 cells were implanted into the rear flank of male athymic mice. Tumors were permitted to form and the animals treated as described in the Methods section. * p < 0.05 less than [pemetrexed + sildenafil] treatment. **E.** NSCLC cells were treated for 12h with vehicle control or [pemetrexed (1.0 μM) + sildenafil (2 μM)] in combination. Cells were then fixed in place and immuno-fluorescence performed to determine the expression levels of PD-L1, PD-L2 and MHCA (changes where noted, green for increased expression red for decreased expression, all p < 0.05).

Finally, we determined whether pemetrexed and sildenafil interacted *in vivo* to suppress lung tumor growth. A transient three-day exposure of established A549 tumors to pemetrexed and sildenafil significantly reduced the rate of tumor growth compared to either drug individually (Figure 13A). This was associated with prolonged animal survival (Figure 13B). Very similar data were obtained using the H460 lung cancer cell line (Figure 13C). Based on our earlier data using an mTOR inhibitor, we determined whether temsirolimus could enhance [pemetrexed + sildenafil] lethality. The growth of A549 cells was reduced by [pemetrexed + sildenafil] that was significantly enhanced by the mTOR inhibitor temsirolimus (Figure 13D). Finally, with a view to performing future additional immunotherapy based studies in immune-competent animals, we determined whether [pemetrexed + sildenafil] altered expression of the immuno-regulatory proteins PD-L1 and PD-L2 in NSCLC cells. A 12h exposure to [pemetrexed + sildenafil] reduced PD-L1 expression whilst that of PD-L2 remained constant (Figure 13E). Expression of MHCA, which would predict for improved NK cell -mediated tumor cell killing, was enhanced by [pemetrexed + sildenafil] drug exposure.

## DISCUSSION

The present studies using the lung cancer chemotherapy drug pemetrexed were based on the foundation of our prior studies combining pemetrexed with the multi-kinase and chaperone inhibitor sorafenib [2, 34]. This concept has moved through phase I evaluation and into phase II for triple negative breast cancer patients [35] (NCT02624700). In contemporaneous parallel studies we had discovered that PDE5 inhibitors in an on-target -dependent fashion could enhance the anti-cancer effects of many standard of care chemotherapeutic drugs, but also of multi-kinase inhibitors such as sorafenib and pazopanib [5–9, 12, 13]. Clinical trials are also open at Massey Cancer Center translating these discoveries with sildenafil (NCT02466802, NCT01817751).

In 2012 Nagai et al demonstrated that increased nitric oxide levels, via cGMP signaling, enhance pemetrexed toxicity in lung cancer cells [36]. Based on our prior data and the results of Nagai et al we attempted to determine whether clinically relevant PDE5 inhibitors such as sildenafil, that in endothelial cells via NO and cGMP counter erectile dysfunction, could through the same signaling pathway enhance pemetrexed toxicity in lung tumor cells. PDE5 inhibitors in a diverse range of lung cancer cell types significantly enhanced pemetrexed toxicity using short-term death assessments and long-term colony formation assays. Although increased signaling through nitric oxide was partially responsible for sildenafil enhancing pemetrexed lethality, knock down of PKGI/II almost abolished the enhancing effect. The precise downstream targets for PKG signaling are likely to be multi-factorial, and our present analyses highlighted that PKG signaling, alongside enhanced signaling by the AMPK, was responsible for the inhibition of chaperone ATPase activities. Reduced chaperone function in a tumor cell would facilitate the inactivation of multiple cyto-protective signaling modules such as the PI3K-AKT-mTOR pathway and lower expression of protective proteins with short half-lives e.g. MCL-1 and c-FLIP-s, as well as facilitating an unfolded protein response that promoted the formation of toxic autophagosomes. Other possible pro-apoptotic targets for PKG signaling include inhibitory phosphorylation of growth factor receptors e.g. c-MET; the inhibition of β-catenin signaling; increasing the expression of 15-LOX-1 with increased synthesis of the bioactive metabolites 13-S-HODE and 15-S-HETE, leading to activation of PPARγ [37–41]. This is of particular note because 15-LOX-1 expression and 13-S-HODE levels are suppressed in lung cancer cells and in other systems, sildenafil has been shown through PKG to regulate PPARγ [42, 43]. It is also known that PPARγ can regulate AMPK and eNOS signaling, which in our system using pemetrexed and sildenafil could be a point of convergence for the observed anti-cancer effects of both drugs [44]. Thus the combination of the Type II diabetes medication rostiglazone with erectile dysfunction drug sildenafil could be a novel way to enhance the anti-tumor effects of maintenance pemetrexed therapy in NSCLC.

Pemetrexed and sildenafil interacted to cause endoplasmic reticulum stress as judged by increased eIF2α serine 51 phosphorylation. Increased eIF2α signaling was essential in the down-regulation of the cyto-protective proteins c-FLIP-s, MCL-1 and BCL-XL, as well as the increase in Beclin1 expression and formation of autophagosomes. However, in contrast to signaling from eIF2α, knock down of IRE1 or XBP-1 enhanced the lethality of [pemetrexed + sildenafil]. Reduced IRE1-XBP-1 signaling increased the basal activities of PERK and eIF2α and reduced the expression of multiple cyto-protective chaperone proteins as well as in a cell-type dependent manner PP1c. These cyto-toxic effect were counteracted by over expression of chaperone proteins. The regulation of GRP78 expression by IRE1-XBP-1 signaling has been established for many years though relatively fewer studies have determined whether this signaling pathway controls the transcription of other chaperone proteins. Nevertheless, XBP-1 signaling has been linked to regulating the expression of GRP94 and HSP40 as well as the p300/PCAF complex which will regulate the function of many genes [45–50].

**Figure 14:**
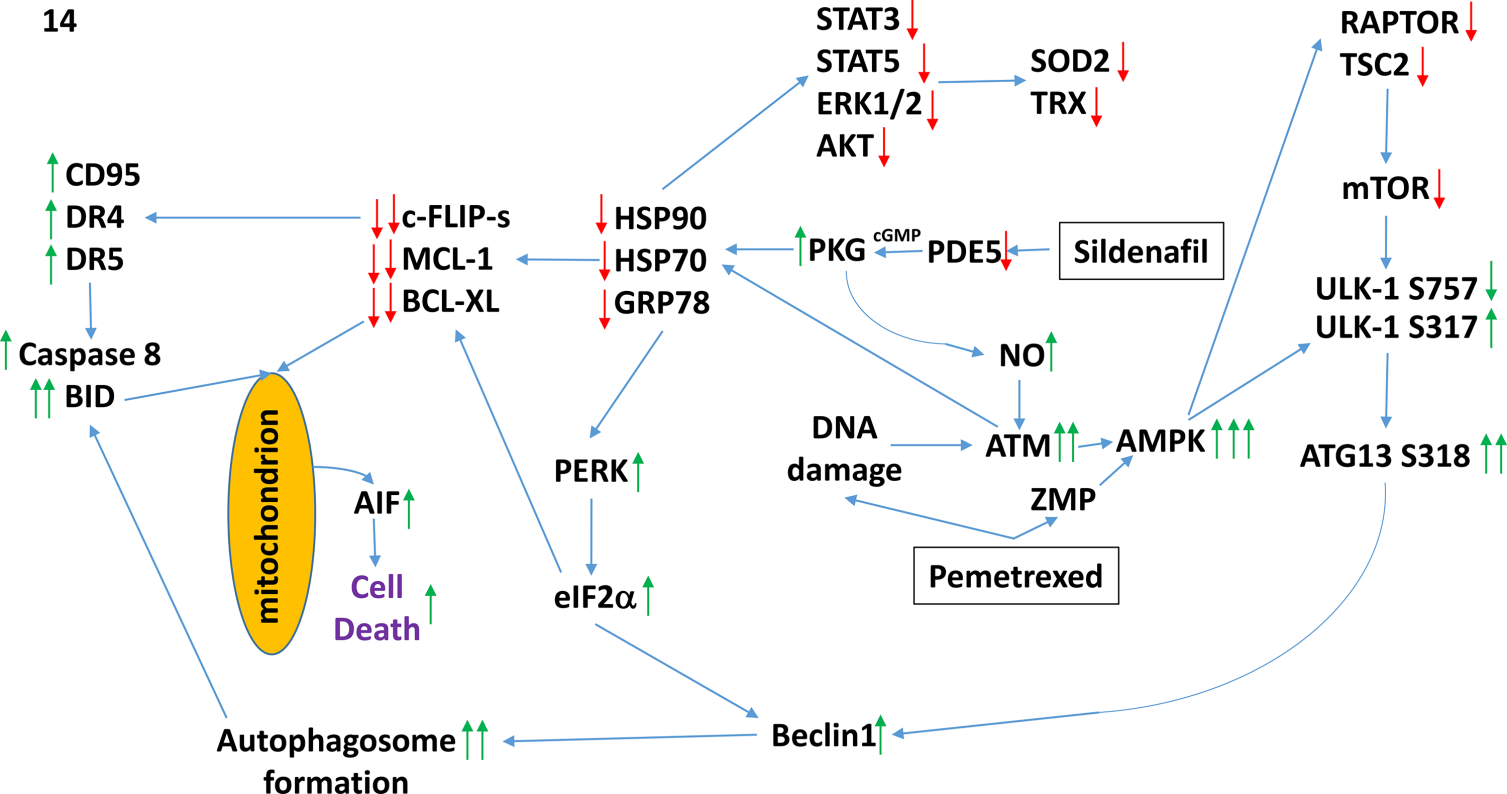
A simplified model of the molecular pathways by which pemetrexed and sildenafil combine to kill lung cancer cells As a thymidylate synthase inhibitor pemetrexed causes DNA damage and increases the levels of ZMP due to inhibition of AICAR. Sildenafil, as a PDE5 inhibitor, increases the levels of cGMP which activates PKG and subsequently leads to the generation of nitric oxide. Nitric oxide enhances the activation of ATM caused initially by DNA damage. ATM signals to activate the AMPK which is further allosterically activated by ZMP. Collectively this strong AMPK signal inactivates RAPTOR and TSC2 resulting in the inactivation of mTORC1 and mTORC2. Downstream of mTOR is the kinase ULK-1; the drug combination via AMPK promotes ULK-1 S317 phosphorylation which activates the kinase; the drug combination via mTOR inactivation reduces ULK-1 S757 phosphorylation which also activates the kinase. Activated ULK-1 phosphorylates ATG13 which is the key gate-keeper step in permitting autophagosome formation. Sildenafil-induced PKG signaling also acts to reduce the activities of multiple chaperone proteins which is augmented by DNA damage induced ATM signaling. Reduced HSP90 and HSP70 function lowers the activities of STAT3, STAT5, ERK1/2 and AKT that results in lower expression of ROS / RNS detoxifying enzymes such as TRX and SOD2. Reduced GRP78 function causes activation of PERK and subsequently eIF2α. Enhanced eIF2α signaling reduces the transcription of proteins with short half-lives such as c-FLIP-s, MCL-1 and BCL-XL, and enhances expression of Beclin1, DR4 and DR5. Thus the convergent actions of reduced HSP90 and HSP70 chaperone activity and eIF2α signaling lead to a profound reduction in the protein levels of c-FLIP-s, MCL-1 and BCL-XL which facilitates death receptor signaling through CD95, DR4 and DR5 to activate the extrinsic apoptosis pathway. Enhanced Beclin1 expression converges with elevated ATG13 phosphorylation to produce high levels of autophagosome formation that, when fused with lysosomes and releasing proteases into the cytosol, converges with the extrinsic apoptosis pathway to cleave BID and cause mitochondrial dysfunction. Tumor cell killing downstream of the mitochondrion was mediated by AIF and not caspases 3/7. The tumoricidal actions of AIF were facilitated by reduced HSP70 functionality as this chaperone can sequester AIF in the cytosol and prevent its translocation to the nucleus.

The molecular mechanisms by which tumor cell death was induced by the drug combination were complex (Figure 14). One component of killing was via increasing the levels of toxic autophagosomes (see below). However, another key component of cell killing was through the activation of extant death receptors (CD95) and PERK-eIF2α-CHOP -dependent increased expression of other death receptors (DR4, DR5). In a cell-type -dependent fashion knock down of CD95, DR4 and DR5 variably prevented the drug combination from killing; knock down of FADD was also protective. Downstream of the receptors caspase 8/10 signaling played a greater role in mediating the death signal than did necroptotic signaling through RIP-1. The PERK-eIF2α-dependent reduction in c-FLIP-s levels played a key role in facilitating death signaling through caspases 8/10. Many investigators over the past 10-15 years have attempted to develop DR4/DR5 as anti-cancer therapeutic targets, e.g. using their natural ligand TRAIL or using DR4/DR5 agonist antibodies. Although TRAIL-DR4/DR5 signaling selectively kills tumor cells over non-transformed cells, the clinical translation of the ligand as a cancer therapeutic has not resulted in significant patient responses. Agonist antibodies against DR4/DR5 are presently being studied in the clinic. Our data would suggest that the combination of [pemetrexed + sildenafil] together with such an agonist antibody may have utility for the treatment of non-small cell lung cancer.

As noted earlier, in parallel to the changes in chaperone expression / function we also found that the combination of [pemetrexed + sildenafil] caused inactivation of the PI3K signaling pathway as judged by reduced phosphorylation of AKT, p70 S6K and mTOR, and activation of AMPK. We discovered that an ATM-AMPK signaling pathway was induced by [pemetrexed + sildenafil] exposure which was responsible for enhancing the mTOR-inhibitory activities of TSC2 and Raptor. Inactivation of mTOR reduces the phosphorylation of the autophagy gate-keeper kinase ULK-1 at serine 757 which promotes kinase activation. Activation of AMPK enhances the phosphorylation of ULK-1 at serine 317 which also promotes kinase activation. Our data argued that for robust ATG13 serine 318 phosphorylation and toxic autophagosome formation to occur, and thus lead to tumor cell death, required both the inactivation of mTOR and the activation of AMPK. Several groups have shown that reactive nitrogen species and reactive oxygen species can activate ATM in the cytoplasm which in turn regulates AMPK that phosphorylates TSC2 and Raptor, inactivating mTOR, leading to a toxic form of autophagy [51, 52]. In glioma cells treated with temozolomide, however, DNA damage activated ATM also signals through AMPK-ULK-1 to promote a protective form of autophagy [53]. And, as pemetrexed increases ZMP levels, the enhanced level of AMPK phosphorylation observed in our system will further stimulate AMPK activity in an allosteric fashion. Thus, [pemetrexed + sildenafil] exposure causes a hyper-activation of the ATM-AMPK pathway through the mechanisms of DNA damage, nitrosative stress and ZMP accumulation which leads to toxic autophagosome formation.

In two models of human NSCLC cells growing in athymic mice we found that pemetrexed and sildenafil interacted in an additive to greater than additive fashion to suppress tumor growth. This effect was further enhanced *in vivo* by the mTOR inhibitor temsirolimus. A549 cells express a mutant active K-RAS and lack expression of p16; H460 cells lack p16, have a mutant active PI3K p110 protein and a mutant active K-RAS. Both cell lines express mutated and truncated LKB1. The H1975 line, used in our *in vitro* studies, expresses a double mutant ERBB1; a mutant PI3K p110 and lacks p16. We have also tested multiple other genetically different NSCLC lines which include lines, e.g. H1573 that expresses a mutant active PI3K p85 protein. From *in vitro* studies using primary non-transformed cells or H&E staining of normal tissues from drug treated mice, as well as examining alterations in mouse body-mass, we conclude that [pemetrexed + sildenafil] treatment is well-tolerated by non-transformed cells and animals under treatment. Unfortunately, Massey Cancer Center has very recently stated to the authors that it is uninterested in translating this concept into the clinic as Massey traditionally has had poor accrual to lung cancer trials and believes it would require too many resources. It is hope other institutions act on the novel data in this manuscript.

## MATERIALS AND METHODS

### Materials

Pemetrexed was purchased from LC Laboratories (Woburn, MA). Sildenafil was purchased from Selleckchem (Houston, TX). Trypsin-EDTA, DMEM, RPMI, penicillin-streptomycin were purchased from GIBCOBRL (GIBCOBRL Life Technologies, Grand Island, NY). Cells were purchased from the ATCC and were not further validated beyond that claimed by ATCC. Cells were re-purchased every ~6 months. ADOR is a primary NSCLC isolate donated to the Dent laboratory by the patient. Spiky are a primary ovarian carcinoma isolate (donated by Dr. Karen Paz, Champions Oncology, NJ). The plasmid to express GRP78/BiP/HSPA5 was kindly provided to the Dent laboratory by Dr. A.S. Lee (University of Southern California, Los Angeles, CA); all other plasmids were purchased from Addgene. Commercially available validated short hairpin RNA molecules to knock down RNA / protein levels were from Qiagen (Valencia, CA) (Figures S2 and S3). Reagents and performance of experimental procedures were described in refs: [1, 2, 6–9].

### Methods

*Culture and in vitro exposure of cells to drugs.* All cell lines were cultured at 37°C (5% (v/v CO_2_) *in vitro* using RPMI supplemented with dialyzed 5% (v/v) fetal calf serum and 10% (v/v) Non-essential amino acids. Cells growing in “complete” fetal calf serum that contains thymidine were gradually weaned into dialyzed serum lacking thymidine over 2 weeks and were then used for experimental analyses for the following 3 weeks before discarding. Cells were re-isolated in thymidine-less media as required. Nota bene: cell killing is reduced by > 50% if exogenous thymidine is supplemented into the growth media. For short term cell killing assays, immunoblotting studies, cells were plated at a density of 3 × 10^3^ per cm^2^ (~2 × 10^5^ cells per well of a 12 well plate) and 48h after plating treated with various drugs, as indicated. *In vitro* pemetrexed, sildenafil and other drug treatments were generally from a 100 mM stock solution of each drug and the maximal concentration of Vehicle carrier (VEH; DMSO) in media was 0.02% (v/v). Cells were not cultured in reduced serum media during any study in this manuscript.

### Transfection of cells with siRNA or with plasmids

#### For Plasmids

Cells were plated and 24h after plating, transfected. Plasmids expressing a specific mRNA (or siRNA) or appropriate vector control plasmid DNA was diluted in 50μl serum-free and antibiotic-free medium (1 portion for each sample). Concurrently, 2μl Lipofectamine 2000 (Invitrogen), was diluted into 50μl of serum-free and antibiotic-free medium (1 portion for each sample). Diluted DNA was added to the diluted Lipofectamine 2000 for each sample and incubated at room temperature for 30 min. This mixture was added to each well / dish of cells containing 200μl serum-free and antibiotic-free medium for a total volume of 300 μl, and the cells were incubated for 4 h at 37°C. An equal volume of 2x medium was then added to each well. Cells were incubated for 24h, then treated with drugs.

#### Transfection for siRNA

Cells from a fresh culture growing in log phase as described above, and 24h after plating transfected. Prior to transfection, the medium was aspirated and serum-free medium was added to each plate. For transfection, 10 nM of the annealed siRNA, the positive sense control doubled stranded siRNA targeting GAPDH or the negative control (a “scrambled” sequence with no significant homology to any known gene sequences from mouse, rat or human cell lines) were used. Ten nM siRNA (scrambled or experimental) was diluted in serum-free media. Four μl Hiperfect (Qiagen) was added to this mixture and the solution was mixed by pipetting up and down several times. This solution was incubated at room temp for 10 min, then added drop-wise to each dish. The medium in each dish was swirled gently to mix, then incubated at 37°C for 2h. Serum-containing medium was added to each plate, and cells were incubated at 37°C for 24h before then treated with drugs (0-24h). Additional immuno-fluorescence / live-dead analyses were performed at the indicated time points.

### Animal studies

Studies were performed according to USDA regulations under VCU IACUC protocol AD20008. Athymic nude mice (~20 g) were injected with 1 × 10^7^ A549 or H460 cells into their rear flank (10 animals per treatment group; 4 groups; a total of 40 mice +/− SEM). Tumors were permitted to form for 7 days with tumors at that time exhibiting a mean volume of ~25 mm^3^. Athymic mice were treated by oral gavage once every day QD for four days as indicated in the Figure and Figure Legend with vehicle (Cremophore); with pemetrexed (50 mg/kg) only on day 1; with sildenafil (5 mg/kg) on days 1-3 and/or with Temsirolimus (10 mg/kg) on Days 1-3. After cessation of drug treatment tumors are again calipered as indicated in the Figure and tumor volume was assessed up to 20-30 days later.

### Detection of cell viability, protein expression and protein phosphorylation by immuno-fluorescence using a Hermes WiScan machine

http://www.idea-bio.com/, Cells (4 × 10^3^) are plated into each well of a 96 well plate, and cells permitted to attach and grow for the next 18h. Based on the experiment, after 18h, cells are then either genetically manipulated, or are treated with drugs. For genetic manipulation, cells are transfected with plasmids or siRNA molecules and incubated for an additional 24h. Cells are treated with vehicle control or with drugs at the indicated final concentrations, alone or in combination. Cells are then isolated for processing at various times following drug exposure. The 96 well plate is centrifuged / cyto-spun to associate dead cells (for live-dead assays) with the base of each well. For live dead assays, after centrifugation, the media is removed and cells treated with live-dead reagent (Thermo Fisher Scientific, Waltham MA) and after 10 min this is removed and the cells in each well are visualized in the Hermes instrument at 10X magnification. Green cells = viable; yellow/red cells = dying/dead. The numbers of viable and dead cells were counted manually from three images taken from each well combined with data from another two wells of separately treated cells (i.e. the data is the mean cell dead from 9 data points from three separate exposures). For immuno-fluorescence studies, after centrifugation, the media is removed and cells are fixed in place and permeabilized using ice cold PBS containing 0.4% paraformaldehyde and 0.5% Triton X-100. After 30 min the cells are washed three times with ice cold PBS and cells are pre-blocked with rat serum for 3h. Cells are then incubated with a primary antibody to detect the expression / phosphorylation of a protein (usually at 1:100 dilution from a commercial vendor) overnight at 37°C. Cells are washed three times with PBS followed by application of the secondary antibody containing an associated fluorescent red or green chemical tag. After 3h of incubation the antibody is removed and the cells washed again. The cells are visualized at either 10X or 60X in the Hermes machine for imaging assessments. All immunofluorescent images for each individual protein / phospho-protein are taken using the identical machine settings so that the levels of signal in each image can be directly compared to the level of signal in the cells treated with drugs. Similarly, for presentation, the enhancement of image brightness/contrast using PhotoShop CS6 is simultaneously performed for each individual set of protein/phospho-protein to permit direct comparison of the image intensity between treatments. Antibodies used include: HSP90 (E289) (Cell Signaling); HSP90 (#2928) (Abcam); HSP90 (ab195575) Abcam; HSP90 3G3 (13495) (Abcam); GRP78 (50b12) (31772) (Cell Signaling); GRP78 (ab191023) Abcam; GRP78 (ab103336) Abcam; GRP78 (N-20) (sc-1050) Santa Cruz; HSP27 (G31) (2402P) Cell Signaling); HSP27 [EP1724Y] (ab62339) Abcam; HSP27 (H-77) (sc-9012) Santa Cruz; HSP27 (LS-C31836) Lifespan science Corp. Other antibodies were as used in prior studies by the laboratory. All immunofluorescent images were initially visualized at 75 dpi using an Odyssey infrared imager (Li-Cor, Lincoln, NE), then processed at 9999 dpi using Adobe Photoshop CS6. For presentation, immunoblots were digitally assessed using the provided Odyssey imager software. Images have their color removed and labeled figures generated in Microsoft PowerPoint.

### The measurement of chaperone ATPase activity

Chaperone ATPase activity using the ATPlite 1step kit (PerkinElmer) was determined using immuno-precipitated HSP90 and HSP70. The Sepharose beads are equilibrated in the reaction buffer provided by the manufacturer for 30 min with gentle mixing, and the beads recovered by centrifugation. The beads are then resuspended 1:1 with reaction buffer. To each well of a 96 well plate is added 50 μl of bead slurry and 50 μl of substrate buffer solution containing vehicle control or drug to achieve the desired final concentration. The reactions are started using a multi-channel pipette delivering 50 μl of reconstituted reagent to each well. The plate is placed in foil in an orbital shaker at 37°C for 15 min. The plate is removed; centrifuged to remove floating Sepharose beads; and 100 μl of the supernatant from each well placed into a new well in another 96 well plate. The light emitted from each well / treatment condition is quantified using a Vector 3 plate reader (n = 3 of three studies +/− SEM).

### Data analysis

Comparison of the effects of various treatments was performed using one-way analysis of variance and a two tailed Student's *t*-test. Statistical examination of *in vivo* animal survival data utilized log rank statistical analyses between the different treatment groups. Differences with a *p*-value of < 0.05 were considered statistically significant. Experiments shown are the means of multiple individual points from multiple experiments (± SEM).

## SUPPLEMENTARY FIGURES



## Abbreviations

ERK: extracellular regulated kinase
MEK: mitogen activated extracellular regulated kinase
PI3K: phosphatidyl inositol 3 kinase
ca: constitutively active
dn: dominant negative
ER: endoplasmic reticulum
mTOR: mammalian target of rapamycin
JAK: Janus Kinase
STAT: Signal Transducers and Activators of Transcription
MAPK: mitogen activated protein kinase
PTEN: phosphatase and tensin homologue on chromosome ten
ROS: reactive oxygen species
CMV: empty vector plasmid or virus
si: small interfering
SCR: scrambled
IP: immunoprecipitation
Ad: adenovirus
VEH: vehicle.
