# Supplementary material for: PDE5 inhibitors enhance the lethality of pemetrexed through inhibition of multiple chaperone proteins and via the actions of cyclic GMP and nitric oxide
Source: Oncotarget. 2016 Nov 26;8(1):1449–68. doi: 10.18632/oncotarget.13640 (PMC5352068; doi:10.18632/oncotarget.13640)
Supplement: Supplementary file 1 [file oncotarget-08-1449-s001.pdf]

PDE5 inhibitors enhance the lethality of pemetrexed through inhibition of multiple chaperone proteins and via the actions of cyclic GMP and nitric oxide

SUPPLEMENTARY FIGURES

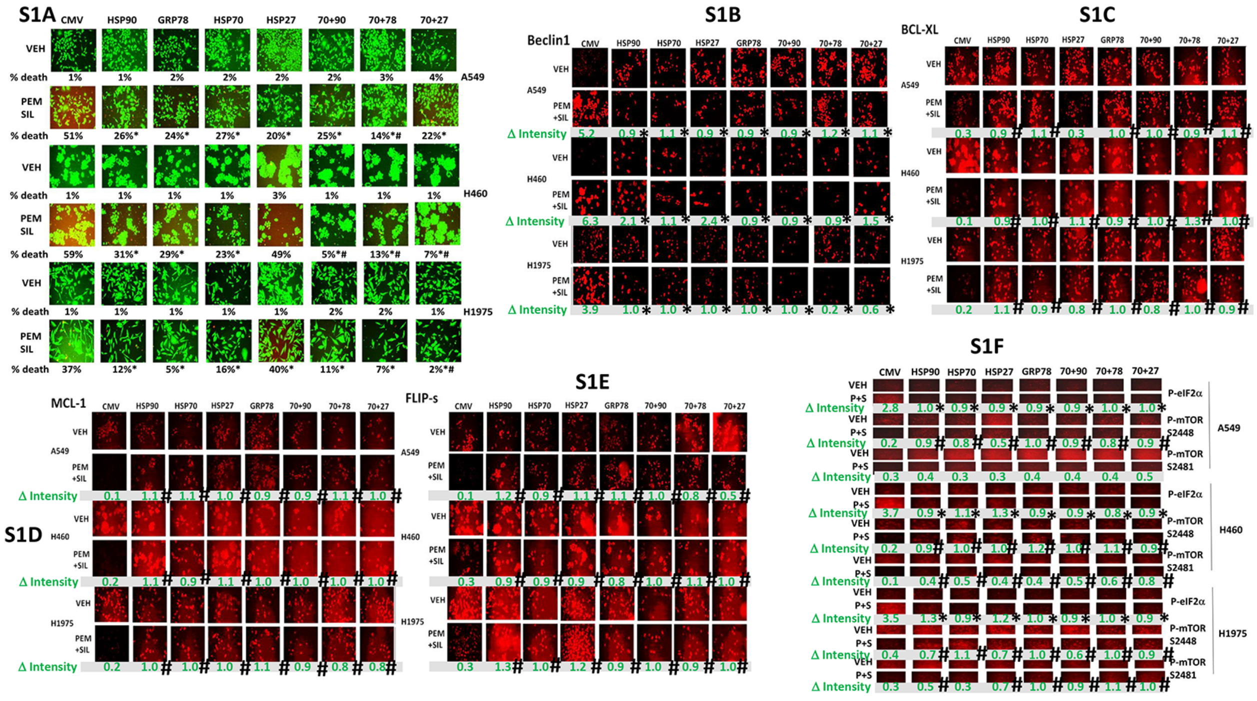

Supplementary Figure S1: Expression of chaperone proteins prevents down-regulation of MCL-1, BCL-XL and c-FLIP-s by [pemetrexed + sildenafil] exposure. A. NSCLC cells were transfected with an empty vector plasmid (CMV) or plasmids to express: HSP90; GRP78; HSP70; HSP27, as indicated. Twenty-four h after transfection cells were treated with vehicle control or [pemetrexed (1.0 µM) + sildenafil (2 µM)] in combination for 24h. Floating cells were cytospun onto the 96 well plate and viability determined using a live / dead viability stain where green cells are viable and yellow / red cells are dead (n = 3 +/- SEM) * $p < 0.05$ less than the corresponding value in CMV cells; # $p < 0.05$ less than corresponding cells transfected to express only one chaperone. **B.E.** NSCLC cells were transfected with an empty vector plasmid (CMV) or plasmids to express: HSP90; GRP78; HSP70; HSP27, as indicated. Twenty-four h after transfection cells were treated with vehicle control or [pemetrexed (1.0 µM) + sildenafil (2 µM)] in combination for 6h. Cells were then fixed in place and immuno-fluorescence staining performed to determine the phosphorylation and expression of the indicated proteins. (n = 3 +/- SEM) * $p < 0.05$ less than the corresponding value in CMV cells; # $p < 0.05$ greater than corresponding value in CMV cells. **F.** NSCLC cells were transfected with an empty vector plasmid (CMV) or plasmids to express: HSP90; GRP78; HSP70; HSP27, as indicated. Twenty-four h after transfection cells were treated with vehicle control or [pemetrexed (1.0 µM) + sildenafil (2 µM)] in combination for 6h. Cells were then fixed in place and immuno-fluorescence staining performed to determine the phosphorylation of: mTOR S2448 (mTORC1); mTOR S2481 (mTORC2); eIF2α S51. (n = 3 +/- SEM) * $p < 0.05$ less than the corresponding value in CMV cells; # $p < 0.05$ greater than corresponding value in CMV cells.

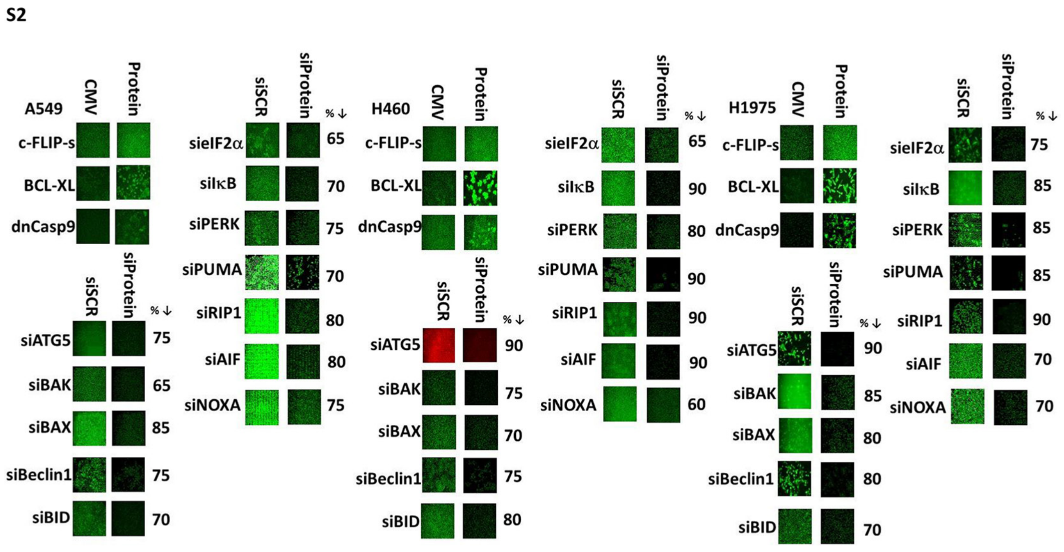

Supplementary Figure S2: Controls to show knock down and over-expression of various proteins examined in the manuscript. The percentage decline in protein expression after siRNA exposure is presented alongside the images showing knock down. Values are rounded to the nearest 5%.

S3

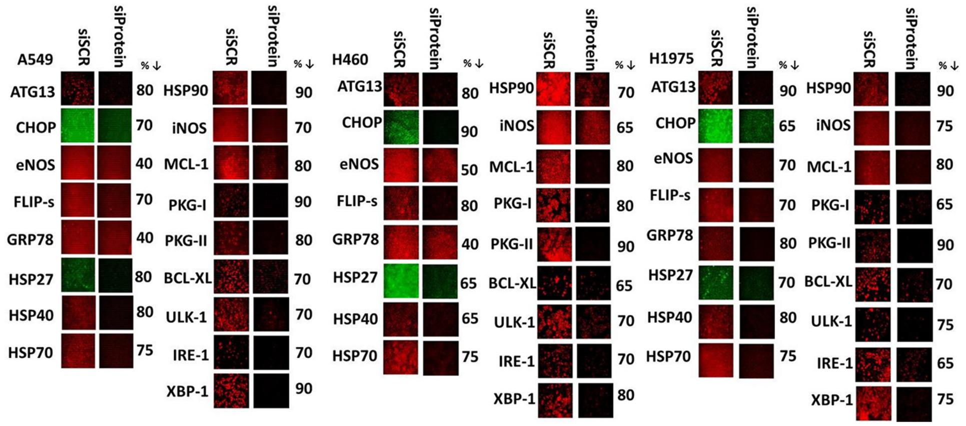

Supplementary Figure S3: Controls to show knock down of various proteins examined in the manuscript. The percentage decline in protein expression after siRNA exposure is presented alongside the images showing knock down. Values are rounded to the nearest 5%.
